# Genome-Wide Analysis of Anthocyanin Biosynthesis Regulatory WD40 Gene *FcTTG1* and Related Family in *Ficus carica* L.

**DOI:** 10.3389/fpls.2022.948084

**Published:** 2022-07-14

**Authors:** Zhiyi Fan, Yanlei Zhai, Yuan Wang, Long Zhang, Miaoyu Song, Moshe A. Flaishman, Huiqin Ma

**Affiliations:** ^1^College of Horticulture, China Agricultural University, Beijing, China; ^2^Department of Fruit Tree Sciences, Agricultural Research Organization, The Volcani Center, Bet Dagan, Israel; ^3^State Key Laboratory of Agrobiotechnology, China Agricultural University, Beijing, China

**Keywords:** fig (*Ficus carica* L.), WD40, expression profile, MBW complex, TTG1, anthocyanin biosynthesis

## Abstract

WD40 proteins serve as crucial regulators in a broad spectrum of plant developmental and physiological processes, including anthocyanin biosynthesis. However, in fig (*Ficus carica* L.), neither the WD40 family nor any member involved in anthocyanin biosynthesis has been elucidated. In the present study, 204 *WD40* genes were identified from the fig genome and phylogenetically classified into 5 clusters and 12 subfamilies. Bioinformatics analysis prediction localized 109, 69, and 26 FcWD40 proteins to the cytoplasm, nucleus and other cellular compartments, respectively. RNA-seq data mining revealed 127 *FcWD40*s expressed at FPKM > 10 in fig fruit. Most of these genes demonstrated higher expression in the early stages of fruit development. FcWD40-97 was recruited according to three criteria: high expression in fig fruit, predicted nuclear localization, and closest clustering with TTG1s identified in other plants. *FcWD40-97*, encoding 339 amino acids including 5 WD-repeat motifs, showed 88.01 and 87.94% amino acid sequence similarity to apple and peach TTG1, respectively. The gene is located on fig chromosome 4, and is composed of 1 intron and 2 exons. Promoter analysis revealed multiple light-responsive elements, one salicylic acid-responsive element, three methyl jasmonate-responsive elements, and one MYB-binding site involved in flavonoid biosynthesis gene regulation. FcWD40-97 was in the FPKM > 100 expression level group in fig fruit, and higher expression was consistently found in the peel compared to the flesh at the same development stages. Expression level did not change significantly under light deprivation, whereas in leaves and roots, its expression was relatively low. Transient expression verified FcWD40-97’s localization to the nucleus. Yeast two-hybrid (Y2H) and biomolecular fluorescence complementation (BiFC) assays revealed that FcWD40-97 interacts with FcMYB114, FcMYB123, and FcbHLH42 proteins *in vitro* and *in vivo*, showing that FcWD40-97 functions as a member of the MYB–bHLH–WD40 (MBW) complex in anthocyanin-biosynthesis regulation in fig. We therefore renamed *FcWD40-97* as *FcTTG1*. Our results provide the first systematic analysis of the *FcWD40* family and identification of *FcTTG1* in fig pigmentation.

## Introduction

WD40 proteins, also called WD-repeat (WDR) proteins, constitute one of the largest protein families in eukaryotic organisms (Stirnimann et al., 2010). WD40 proteins were first defined in bovine β-transducin, with an approximately 43-residue fragment containing a glycine–histidine (GH) pair at the N terminus and a tryptophan–aspartate (WD) pair at the C terminus (Fong et al., 1986). WD40 proteins are usually regarded as adaptors, recruiting other factors to form protein complexes or protein–DNA complexes (Jain and Pandey, 2018; Dayebgadoh et al., 2019). There is accumulating evidence of WD40s’ versatile roles in a wide spectrum of biochemical, developmental and physiological processes, including signal transduction, chromatin assembly, cytoskeleton assembly, cell division, flowering and abiotic stress responses (Smith, 2008; Hu et al., 2020; Tan et al., 2021).

WD40s have been grouped in different ways by different researchers, according to evolutionary relationships or comparisons with *Arabidopsis*. In *Arabidopsis thaliana*, 237 *AtWD40* genes with at least 4 WDR motifs were classified into 33 groups, and in *Cucumis sativus*, 191 *CsWD40* genes were classified into 21 groups (Li et al., 2014). In *Solanum tuberosum*, 178 *StWD40* genes and in *Mangifera indica*, 315 *MiWD40* genes were classified into 14 and 11 groups, respectively (Liu et al., 2020; Tan et al., 2021). In *Triticum aestivum*, *Prunus persica*, *Rosa chinensis*, and *Ginkgo biloba*, 743 *TaWD40* genes, 220 *PpWD40* genes, 187 *RcWD40* genes, and 167 *GbWD40* genes were each clustered into 5 groups (Hu et al., 2018; Feng et al., 2019; Sun et al., 2020; Zheng et al., 2021a,b).

The most well-known function of WD40 is as part of the MYB–bHLH–WD40 (MBW) complex in the regulation of anthocyanin/proanthocyanidin biosynthesis. TRANSPARENT TESTA GLABRA1 (TTG1) is a WD40 protein that was first identified in *Arabidopsis*; *AtTTG1* encodes 341 amino acids; *Atttg1* mutants completely lack anthocyanins in the epidermis and in subepidermal layers of leaves and stems (Walker et al., 1999). Moreover, AtTTG1, AtMYB123, and AtbHLH42 can form a ternary complex that synergistically activates the expression of *BANYULS* (anthocyanidin reductase) and promotes proanthocyanidin biosynthesis (Baudry et al., 2004). *TTG1* has been identified in other plants as well; *Fragaria* × *ananassa* (strawberry) *FaTTG1*, *Camellia sinensis CsWD40* and *Vitis vinifera* (grape) *VvWDR1* encode 344, 342, and 336 amino acids, respectively (Schaart et al., 2013; Liu et al., 2018; Jiu et al., 2021). Overexpression of *TTG1-like CsWD40* in tobacco resulted in a significant increase in anthocyanin content in the petals of the transgenic plants; when it was coexpressed with *CsMYB5e* in tobacco, both anthocyanin and proanthocyanidin content increased (Liu et al., 2018). VvWDR1 increased anthocyanin accumulation in transgenic tobacco by interacting with the VvMYBA2r–VvMYCA1 complex (Jiu et al., 2021).

Fig (*Ficus carica* L.) is one of the most ancient cultivated fruit trees in the world. Fig fruit development presents a typical double-sigmoid growth curve, i.e., a fast–slow–fast growth pattern (Kislev et al., 2006; Stover et al., 2007; Flaishman et al., 2008). Fig fruit have a high content of anthocyanins, proanthocyanidins and other flavonoids (Solomon et al., 2006; Wang et al., 2019). The pigmentation of fig peel and flesh tissue follows an obvious tissue-specific pattern. The fig peel can be green, yellow or red/black-purple at ripening, whereas the flesh can be amber or from light to deep red at harvest.

A series of anthocyanin biosynthesis-related genes have been functionally characterized in fig, including *FcANS* (Cao et al., 2016), *FcMADS9* (Li et al., 2021) and *FcHY5* (Wang et al., 2019); in addition, three *MYB* genes—*FcMYB21*, *FcMYB114* and *FcMYB123* (Wang et al., 2019; Li et al., 2020)—and bHLH regulator *FcbHLH42* (Song et al., 2021) were also functionally proven to participate in anthocyanin and flavonoid biosynthesis. Although a *FcWD40* gene was isolated (Lama et al., 2020), there has been no systematic analysis of the *FcWD40* gene family, nor has *FcTTG1* been identified. Based on the published fig genome (Usai et al., 2020), we screened 204 putative *FcWD40* genes and studied their structure, cellular localization and expression patterns in fig fruit. *FcWD40-97* was identified by closest sequence similarity to TTG1s from other plants and its interaction with fig anthocyanin-biosynthesis regulators FcMYBs and FcbHLH. The result provides us with the first global view of FcWD40s and reveals *TTG1* in fig.

## Materials and Methods

### Search for *FcWD40*s

The whole-genome sequence of fig was downloaded from the NCBI online database (^1^ Usai et al., 2020); data of *Ficus microcarpa* and *Ficus hispida* were downloaded from the Genome Warehouse (GWH) and Sequence Archive (GSA) database (Zhang et al., 2020). Protein data of *A. thaliana* Araport11 were downloaded from the TAIR database^2^. The Hidden Markov Model (HMM) profile of the WD40 domain (PF00400) was retrieved from Pfam^3^. Candidate genes with typical WD40 domains in the fig genome were preliminarily screened using the HMM and SMART online tools (Eddy, 1998; Letunic and Bork, 2018). Systematic WD40 domain (PF00400) analysis was performed using the Pfam database (Finn et al., 2014). The redundant sequences were deleted utilizing HMMER software^4^ with a default E-value < 10^–5^, then sequences with less than 150 amino acids were discarded; this was followed by a high-standard (E-value < 10^–10^) screening of the conserved WD40 domain using NCBI CDD^5^. Finally, all of the candidate sequences were confirmed by Pfam and the conserved domain database (Marchler-Bauer and Bryant, 2004).

### Bioinformatics Analysis

ClustalW software with default parameters was used to carry out multiple-sequence alignments of the predicted WD40 protein sequences of fig and *Arabidopsis* (Thompson et al., 2002). An unrooted phylogenetic tree was constructed using the neighbor-joining method by MEGA 7 with the following parameters: Poisson model, pairwise deletion, 1,000 bootstrap iterations.

The coding sequences and genomic sequences of the identified *FcWD40* genes were obtained. The location of each *FcWD40* on the 13 fig chromosomes was determined by searching *WD40* sequences to fig chromosome using Blast programs. An intron/exon structure map of the *FcWD40*s was drawn using TBtools (Chen et al., 2020). The ExPASy Server tool^6^ was applied to predict the molecular mass and theoretical isoelectric point (pI) of the WD40 proteins. The PSORT II database^7^ was used to examine the subcellular localization of FcWD40 proteins. In addition, the WD40 domain and motif were identified in the genes.

MCScanX was used to analyze synteny of the *WD40* genes between fig and other plants (Wang et al., 2012), and collinearity relationship was drawn by Circos and TBtool software (Krzywinski et al., 2009). Ka/Ks was calculated with KaKs Calculator 2.0^8^ (Wang et al., 2010). The gene duplications were dated using the formula T = Ks/2r; r, which is the rate of divergence for nuclear genes, was taken to be 7 × 10^–9^ synonymous substitutions per site per year according to a previous report on *A. thaliana* (Ossowski et al., 2010). The potential *cis*-regulatory elements on promoter regions of *WD40* genes were identified by Plant CARE (Lescot et al., 2002).

### RNA-Seq Data Mining

Two fig fruit RNA-seq libraries which were established and uploaded to NCBI by our laboratory were re-mined. PRJNA723733^9^ is a library of fruit flesh and peel at six stages covering the whole fruit development process of cv. “Purple-Peel,” which has a purple peel and red flesh (Song et al., 2021). PRJNA494945^10^ is a transcriptome library of light-deprived “Purple-Peel” fig fruit and a control (Wang et al., 2019). The number of mapped clean reads for each transcript was normalized to FPKM (fragments per kilobase of exon model per million mapped reads) by RSEM^11^. Genes that were differentially expressed under light deprivation and their corresponding controls were analyzed by edgeR^12^ (Robinson et al., 2010).

### Cloning of *FcTTG1* and qRT-PCR Analysis

Fig root, shoot, young leaf, old leaf and fruit were collected from greenhouse-grown “Purple-Peel” fig trees at the Shangzhuang Experimental Station of China Agricultural University at Beijing. The fig trees were planted with 3.5 m × 4 m spacing in 2017. Fig fruit were separated into peel and flesh using a scalpel. All of the plant material was frozen using liquid nitrogen and stored for later use. DNA was extracted from fig leaves using the cetyltrimethylammonium bromide (CTAB) method (Murray and Thompson, 1980), and RNA was isolated following protocols developed in our laboratory and used in previous publications (Chai et al., 2014).

The *FcTTG1* gene sequence was cloned from ‘Purple-Peel’ fig flesh. The expression of *FcWD40-97* was validated by qRT-PCR. PrimeScript™ RT reagent kit (RR037Q, Takara, Dalian, China) was used to reverse-transcribe the total RNA. *18S* was used in parallel as an internal reference gene to normalize the expression levels. The qRT-PCR was performed with ChamQ Universal SYBR qPCR Master Mix (Q711-02, Vazyme, Nanjing, China). A 10-μL reaction mixture was added to each well. The PCR program was as follows: 95°C for 30 s, and 40 cycles of: 95°C for 10 s, 60°C for 60 s. Each sample was analyzed in three technical replicates, and the 2^–ΔΔCt^ method was applied for relative quantification analysis (Livak and Schmittgen, 2001). The expression of each gene was determined using three biological replicates. Statistical analysis of one-way ANOVA followed by Duncan’s new multiple range test and Student’s *t*-test were performed with IBM SPSS Statistics Version 23.0. The significance level was set to *P* < 0.05. All primers used for qRT-PCR are listed in Supplementary Table 1.

### Subcellular Localization and Prediction of Protein–Protein Interactions for FcTTG1

The full-length cDNA of *FcTTG1* was cloned for subcellular localization using primers containing restriction sites *Xba*I and *Bam*HI, and inserted into binary vector pBI121 to generate fusion construct 35S:FcTTG1-GFP. Then, 35S:FcTTG1-GFP and the control vector were transferred into *Agrobacterium tumefaciens* strain GV3101 by heat shock. Four-week-old *Nicotiana tabacum* leaves were agroinfiltrated (Kumar and Kirti, 2010) with the bacterial suspension (OD_600_ = 0.8) and then kept in an incubator at 28°C for 72 h, followed by live-cell imaging using an inverted confocal laser-scanning microscope (Olympus FV3000, Tokyo, Japan).

The STRING 11.5 server^13^ was employed to illustrate the protein–protein interaction network. The exhibited specifications were used with confidence values of 0.4 for network, 10 for edge evidence and maximum number of interactions.

### Yeast Two-Hybrid and Bimolecular Fluorescence Complementation Assays

The interaction of FcTTG1 with FcMYBs and FcbHLHs was explored using the Matchmaker Gold Yeast Two-Hybrid System (Clontech, USA). *FcTTG1* was ligated to a pGBKT7 vector used as bait, and *FcMYB114*, *FcbHLH1*, *FcbHLH42*, and *FcMYC2* were ligated to a pGADT7 vector and used as prey proteins. Plasmids pGBKT7-53 plus pGADT7-T and pGADT7-lam plus pGADT7-T plasmids were used as positive and negative controls, respectively. Yeast cells were cultured on the SD/-Leu-Trp (DDO) medium, then transformed colonies were plated on SD/-Leu-Trp-His-Ade (QDO) with 20 mM X-α-gal and 125 ng/mL AbA medium for further verification of the actual interactions. The yeast strains were co-transformed with *FcTTG1* plus *FcMYB114*, *FcMYB123*, *FcbHLH1*, *FcbHLH42*, and *FcMYC2*, respectively. The primers used for Y2H vector construction are presented in Supplementary Table 1.

The full-length cDNA of *FcTTG1* was inserted into a pSPYCE-35S (YCE) vector, and those of *FcMYB114*, *FcMYB123* and *FcbHLH42* were respectively subcloned into pSPYNE-35S (YNE) vectors. All of the constructs and control vectors (pSPYNE-35S and pSPYCE-35S) were transformed into *A. tumefaciens* strain GV3101, and 4-week-old *N. benthamiana* plants leaves were transiently co-transformed as described by Song et al. (2022). Fluorescence signals were observed 72 h after transfection using a confocal laser-scanning microscope (Olympus FV3000, Tokyo, Japan). The primers used for the BiFC vector construction are presented in Supplementary Table 1.

## Results

### Phylogenetic Analysis

A total of 204 WD40 proteins with at least 1 WDR motif were recruited from *F. carica* after removing repetitive sequences. For convenience, the genes were numbered from *FcWD40-1* to *FcWD40-204*. Among them, 190 genes had at least 4 WDR motifs. FcWD40 proteins varied from 189 (FcWD40-204) to 3628 (FcWD40-28) amino acids, and the predicted molecular mass ranged from 20.67 kDa (FcWD40-204) to 403.52 kDa (FcWD40-28). Theoretical pI ranged from 4.29 to 10.46, with 120 acidic and 84 alkaline pI. Predicted subcellular localization showed 109 FcWD40 proteins in the cytoplasm, 69 in the nucleus, 10 in the endoplasmic reticulum, 10 in the mitochondria, 3 in the plasma membrane, 2 in the Golgi, and 1 in the vacuole (Supplementary Table 2).

**FIGURE 1:**
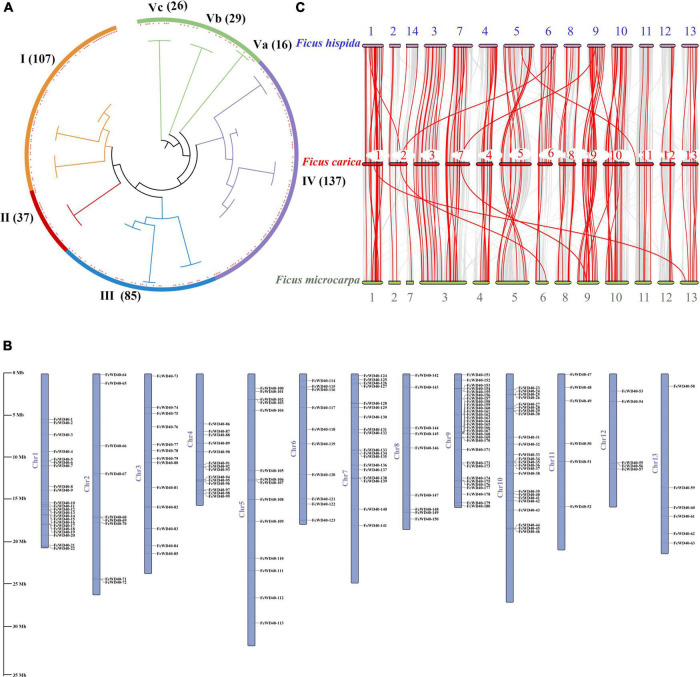
Chromosomal locations, phylogenetic relationships, and collinearity analysis of the *FcWD40* gene family. **(A)** Phylogenetic tree of 437 *WD40* genes identified in *Ficus carica* and *Arabidopsis thaliana* genomes. Orange, red, blue, purple, and green highlighted areas show clusters I–V. Red squares represent fig WD40. The numbers on the branch points represent total number of WD40 gene; scale length represents genetic distance. **(B)** *FcWD40* genes are marked on chromosomes; scale bar on the left indicates length of fig chromosome (Mb). **(C)** Collinearity relationship of *WD40* genes among *Ficus carica*, *Ficus hispida*, and *Ficus microcarpa*. Identified collinear genes are linked by red lines.

A phylogenetic tree was generated using the 204 FcWD40s and 233 AtWD40s. The FcWD40s were categorized into five clusters (I–V), and cluster V had three subclusters (Va, Vb, and Vc). Cluster IV was the largest subgroup with 137 members, followed by cluster I with 107 members; cluster II was the smallest, with 37 members (Figure 1A).

### Chromosomal Distribution and Evolution

We mapped 180 *FcWD40*s to 13 chromosomes; positions of the other 24 genes were not found. *FcWD40*s were widespread and unevenly distributed on the fig chromosomes (Figure 1B). Chromosome 3 hosted 30 *FcWD40*s, followed by chromosome 9 with 24 genes; only 5 genes were mapped to chromosome 12. Three pairs of genes—*FcWD40-15*/*FcWD40-16*, *FcWD40-44*/*FcWD40-45*, *FcWD40-154*/*FcWD40-155*—were identified as tandem duplications, and eight pairs of genes might have resulted from segmental duplication events (Supplementary Figure 1).

The substitution rate of non-synonymous (Ka) and synonymous (Ks) is the basis for evaluating the positive selection pressure of duplication events. We identified the eight duplicated gene pairs in the *FcWD40* family (Table 1). The Ka/Ks ratios of the eight duplicated gene pairs ranged from 0.08 to 0.71, suggesting negative selective pressure on them. Duplication of these eight *WD40* gene pairs was calculated to have occurred between 0.32 and 209.82 million years ago.

**TABLE 1:** Ka/Ks calculation and divergence time of the duplicated *FcWD40* gene pairs.

Duplicated pair	Ka	Ks	Ka/Ks	Divergence time (MYA)
FcWD40-9/FcWD40-20	0.0032	0.0045	0.7113	0.32
FcWD40-4/FcWD40-175	0.1229	1.5013	0.0819	107.24
FcWD40-43/FcWD40-46	0.0419	0.09	0.4661	6.43
FcWD40-31/FcWD40-56	0.3264	2.9375	0.1111	209.82
FcWD40-76/FcWD40-77	0.0445	0.0518	0.859	3.7
FcWD40-99/FcWD40-138	0.3476	1.7944	0.1937	128.17
FcWD40-113/FcWD40-130	0.3303	1.4407	0.2293	102.91
FcWD40-173/FcWD40-174	0.0023	0.0074	0.313	0.53

Comparative syntenic maps were constructed using *FcWD40*s associated with those of *F. hispida* and *F. microcarpa* to reveal phylogenetic relationships between species (Figure 1C). The collinearity analysis identified 184 and 206 WD40 orthologs in *F. hispida* and *F. microcarpa*, respectively; 110 *FcWD40* genes showed a collinear relationship with those of the two other *Ficus* species, whereas 34 *FcWD40* genes did not, suggesting unique *WD40*s in the evolution of *F. carica*. Further analysis demonstrated that *F. hispida WD40* genes had the most homologous gene pairs with the *WD40* genes of *F. carica* (134), followed by *F. microcarpa* (125), indicating similar evolutionary distances between the edible fig and the two evergreen *Ficus* species. Moreover, in comparison with *F. microcarpa*, *F. hispida* genes showed a closer phylogenetic relationship with *FcWD40* genes. Detailed results of the analysis are shown in Supplementary Table 3.

### Gene Structure and Promoter Analysis

**FIGURE 2:**
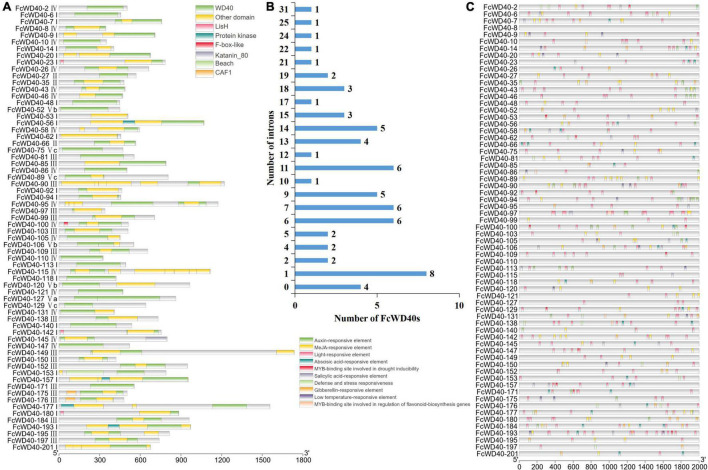
Analysis of domain structure and promoter of FcWD40s. **(A)** Distribution of primary domains of 66 FcWD40 proteins with nuclear localization and genes expressed in fig fruit. **(B)** Introns number of 66 FcWD40s. **(C)** Promoter analysis of *FcWD40*s by Plant CARE, based on 2000-bp sequence upstream of the genes.

FcWD40s were categorized into 12 subfamilies based on their domain compositions. Sixty-three *FcWD40* genes containing only WD40 domains were classified as subfamily A; among them, 23 had predicted nuclear localization. Eighty-seven *FcWD40* genes containing another unknown domain were classified as subfamily L, among them 34 genes were with predicted nuclear localization. The remaining 54 *FcWD40*s, comprising other domains, were grouped into subfamilies B, comprising other 2 genes containing the zinc finger domain; (C) 4 genes with the CAF1 domain, including 2 genes with predicted nuclear localization; (D) 16 genes with the LisH domain, including 4 genes with predicted nuclear localization; (E) 4 genes with the UTP12/UTP15/UTP21 domains; FcWD40s with (F) the coatomer WD/COP1 domain, (G) katanim_80, (H) beach, (I) protein kinase, (J) NLE, and (K) F-box-like domain. Transcription regulation occurs in the nucleus, and at least 4 WDR motifs are required to form a β-propeller (Chothia et al., 1997). Therefore, the 66 FcWD40s with predicted nuclear localization and with at least 4 WDR motifs were selected for analysis; their domains are shown in Figure 2A, and a phylogenetic tree was generated (Supplementary Figure 2).

Gene structure analysis of the *FcWD40*s revealed wide variation in the number of exons and introns. The maximum number of predicted exons, 40, was found in *FcWD40-128*, whereas only 1 exon and no intron were found in 9 *FcWD40* genes (Supplementary Table 2). The gene structures of the aforementioned 66 *FcWD40s* were also examined. Among them, *FcWD40-8*, *FcWD40-48*, *FcWD40-113*, and *FcWD40-118* had no intron, whereas the other 62 *FcWD40s* had 1–31 introns (Figure 2B).

Ten conserved motifs were identified in the FcWD40 proteins, designated motif 1 to 10. Motifs 1–5 were identified as the WD40 domain. All 204 FcWD40 proteins had motif 1, which contained 15 amino acids, and 202 FcWD40 proteins had motif 2; 19 FcWD40 proteins had motif 3; 198 and 197 FcWD40 proteins had motif 4 and motif 5, respectively. Eight FcWD40 proteins had motif 6 or motif 7. Five FcWD40 proteins had motif 8, and motifs 9 and 10 were the least common (Supplementary Table 4).

A variety of *cis*-elements were revealed in the 2-kb promoter region of the 66 *FcWD40*s with predicted nuclear localization. *Cis*-elements related to hormones, such as auxin, methyl jasmonate (MeJA), abscisic acid, salicylic acid and gibberellin were rather common, suggesting that *FcWD40* gene expression could be responsive to a variety of plant hormones. Furthermore, light, defense and stress, low temperatures, and a MYB-binding site involved in drought inducibility were identified (Figure 2C). Notably, only *FcWD40-97* had a MYB-binding site involved in the regulation of flavonoid gene biosynthesis among the 66 putatively nuclear-localized *FcWD40* genes, and it had 3 MeJA-responsive elements as well (Figure 2C).

### Expression Pattern in Fruit Development

**FIGURE 3:**
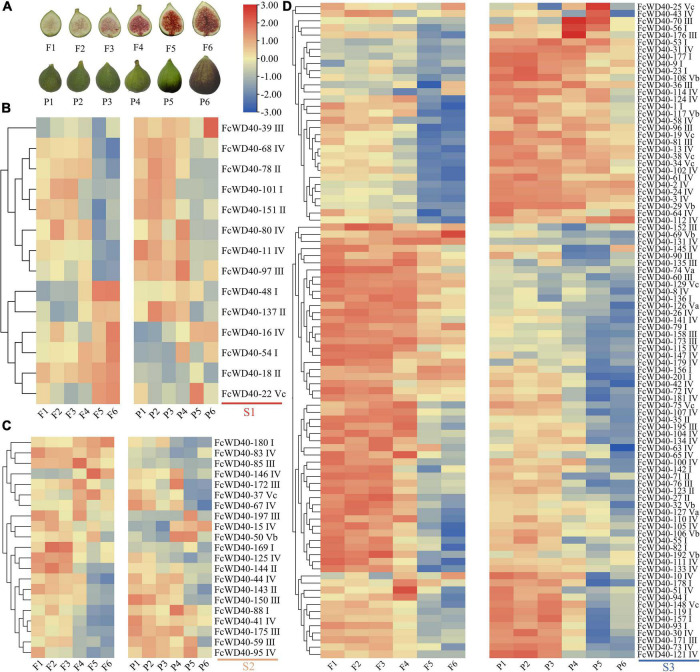
Expression profiles of *FcWD40* genes in the flesh and peel tissue of developing and ripening fruit. **(A)** Photographs of fig flesh and peel color at different stages. **(B)** Members of FcWD40 in group S1 (FPKM ≥ 100). **(C)** Members of FcWD40 in group S2 (100 > FPKM ≥ 50). **(D)** Members of FcWD40 in group S3 (50 > FPKM > 10). Color bars represent level of downregulation (blue) and upregulation (red).

One-hundred and twenty-seven *FcWD40*s were expressed at FPKM > 10 in at least one of the six samples covering the whole fig fruit development process. Three expression groups (S1–S3) were assembled based on the genes’ highest transcription levels: S1, at least one sample of FPKM ≥ 100; S2, FPKM between 99 and 50; and S3, FPKM between 49 and 10. There were 14, 21, and 92 *FcWD40*s in groups S1, S2, and S3, respectively (Figure 3). In group S1, *FcWD40-11/68/78/80/97/101* were highly expressed in stages 1–4, while *FcWD40-16/18/22/54* were significantly upregulated in stages 5 or 6, in both flesh and peel.

Different expression patterns of *FcWD40*s were found in the peel and flesh. *FcWD40-48* and *FcWD40-137* were highly expressed in stage 5 and 6 in the flesh, but had lower expression levels in the corresponding peel samples. *FcWD40-39* was specifically expressed in the peel. Most of the group S2 and S3 *FcWD40*s had higher expression in stages 1–4 (Figure 3). Of the 66 FcWD40s predicted to be located in the nucleus, 43 showed expression of FPKM > 10 in fig fruit. These included *FcWD40-48* and *FcWD40-97* in group S1, 6 genes: *FcWD40-85/95/150/175/180/197* in group S2, and 35 genes in group S3.

**FIGURE 4:**
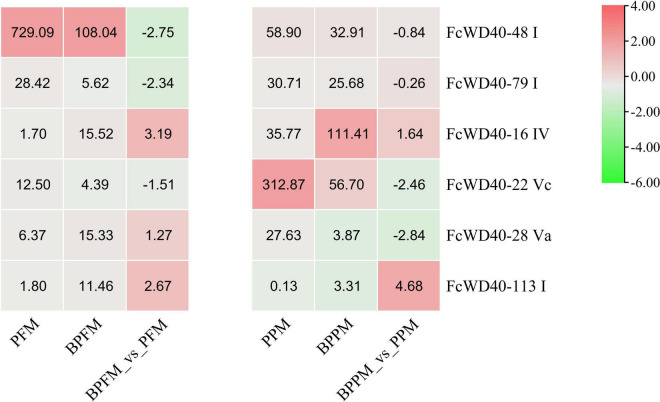
Expression profiles of *FcWD40* genes under light deprivation. PFM and PPM represent the mature-stage flesh and peel of “Purple-Peel,” respectively; BPFM and BPPM represent the bagging mature-stage flesh and peel of ‘Purple-Peel’ fruit, respectively.

Six *FcWD40*s in fig fruit showed a significant change in expression under light deprivation (FPKM > 10, | log2(fold change)| ≥ 2, *p*-adjust < 0.05): *FcWD40-22* and *FcWD40-28* showed differential expression in the peel; *FcWD40-16*, *FcWD40-48*, and *FcWD40-79* showed differential expression in the flesh, respectively, moreover, *FcWD40-113* showed differential expression in both tissue types (Figure 4). Among these 6 *FcWD40s*, *FcWD40-48* and *FcWD40-113* were predicted to be located in the nucleus; 3 of the 6 *FcWD40*s were from group S1, i.e., 11 members of group S1 *FcWD40*s did not respond to fruit light deprivation.

### Screen for FcTTG1 and Expression Analysis

**FIGURE 5:**
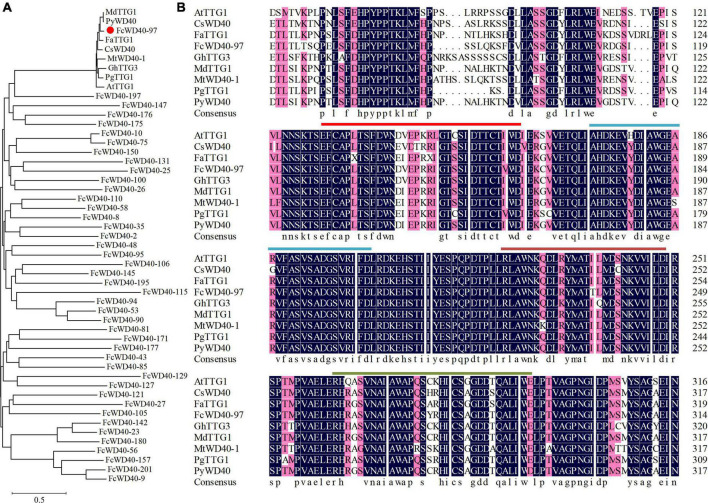
Phylogenetic analysis of 43 FcWD40 proteins and TTG1s of other species. **(A)** Phylogenetic relationships of TTG1 proteins. GenBank accession numbers: *Arabidopsis thaliana AtTTG1* (NM_122360), *Malus domestica MdTTG1* (GU173813), *Pyrus pyrifolia PyWD40* (HQ641374.1), *Fragaria* × *ananassa FaTTG1* (JQ989287), *Camellia sinensis CsWD40* (MH618664), *Punica granatum PgTTG1* (HQ199314), G*ossypium hirsutum GhTTG3* (AAM95645), and *Medicago truncatula*, *MtWD40*-1 (EU040206.1). *FcWD40-97* is indicated with red dot. **(B)** Alignment of deduced amino acid sequences of FcWD40 proteins and other known TTG1 homologs. The four conserved amino acid residue pairs (WD, FD, LD, and WE) are marked by different-colored straight lines.

To find the WD40s associated with fig fruit color development, a phylogenetic tree was constructed using the 43 fruit-expressed *FcWD40* genes with predicted nuclear localization and with at least 4 WDR motifs, together with 8 TTG1 proteins identified from other plants (Figure 5A). Another phylogenetic tree was constructed to further analyze the evolutionary relationships of all FcWD40s and 8 TTG1 proteins (Supplementary Figure 3). FcWD40-97 showed the highest sequence similarity to the TTG1s. The deduced amino acid sequence of FcWD40-97 was 79.88% similar to TTG1 from *Arabidopsis*, 88.01 and 87.94% similar to apple and peach TTG1, respectively, and 85.67 and 84.88% similar to CsWD40 and FaTTG1, respectively. The four hypothetical amino acid residue pairs (WD, FD, LD, and WE) were also highly conserved among the TTG1 proteins involved in anthocyanin or proanthocyanidin biosynthesis in different plants (Figure 5B). Therefore, we suggested that FcWD40-97 is a FcTTG1.

**FIGURE 6:**
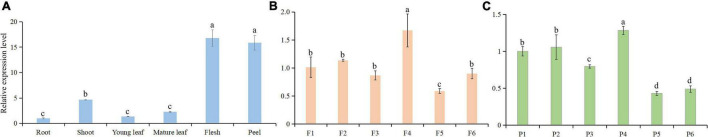
Relative expression levels of *FcTTG1* in different tissues and at different developmental stages in *Ficus carica*. **(A)** Fig plant tissues; flesh and peel are from ripe fruit. **(B)** Fig fruit flesh at developmental stages 1–6 corresponding to Figure 3A. **(C)** Fig fruit peel at developmental stages 1–6 corresponding to Figure 3A. Relative expression levels are shown as mean ± SD from three replications. Different letters between bars indicate significant difference at *p* < 0.05.

Expression of *FcWD40-97* in different tissues of the fig tree was determined by qRT-PCR. It was expressed in all six tissues examined, with significantly higher transcript levels in the flesh and peel, followed by shoots; its expression level in young and mature leaves and roots was markedly lower (Figure 6A). In the fruit, *FcWD40-97* was more highly expressed at developmental stage 4 in both flesh and peel (Figures 6B,C), which was largely consistent with our fig fruit RNA-seq results.

### Subcellular Localization and Interaction Network of FcWD40-97

**FIGURE 7:**
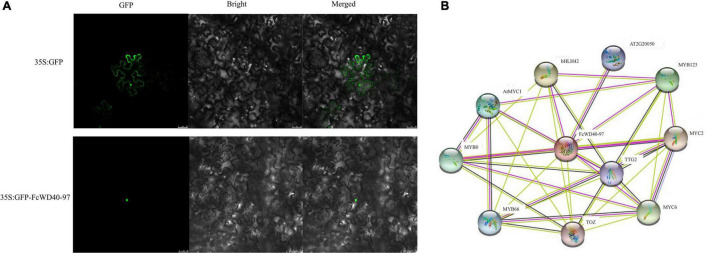
Subcellular localization and interaction network prediction of FcTTG1. **(A)** Subcellular localization of FcTTG1 in *Nicotiana benthamiana*. Images under fluorescence (left), bright field (middle) and merged (right). Bars = 50 μm. **(B)** Predicted interaction network for FcTTG1. Pink lines – experimentally determined physical interactions; yellow lines – interactions by text mining; black lines – co-expression; purple lines – protein homology.

The predicted nuclear localization of FcWD40-97 was validated by fusing its full-length ORF to the N terminus of green fluorescent protein (GFP) driven by the CaMV 35S promoter. By fluorescence microscopy, the 35S:FcTTG1–GFP fusion proteins were detected exclusively in the cell nucleus, whereas the control was uniformly dispersed throughout the cell (Figure 7A).

Computational analysis of protein–protein interactions was performed to predict FcWD40-97’s function. The interaction network was observed among several proteins, including FcWD40-97’s interaction with 4 bHLH proteinsork was observed among several proteins, including predict FcWD40-97’s function. detected exclusively in, AT2G20050 (PP2C) and TOZ (WD40), depicting the physical interactions, co-expression, and homology of the proteins (Figure 7B).

**FIGURE 8:**
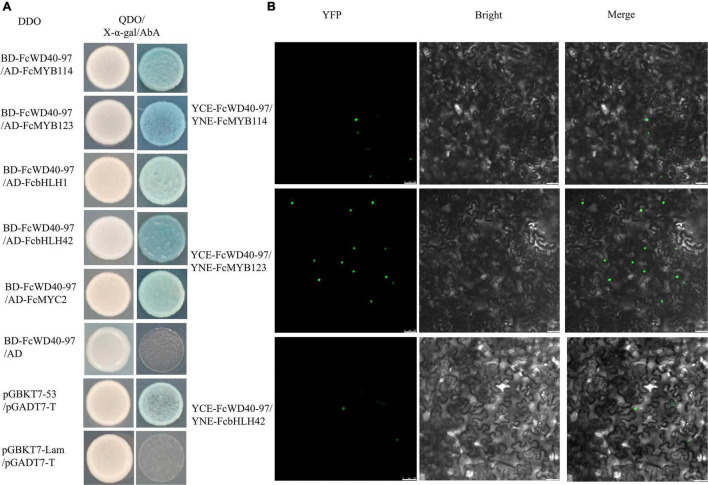
Interaction of FcTTG1 with FcMYBs and FcbHLHs *in vitro* and *in vivo*. **(A)** Yeast two-hybrid test to determine the *in vitro* interaction with different constructs. **(B)** *In vivo* verification of interactions between FcTTG1 and FcMYB114, FcMYB123, and FcbHLH42. Bars = 50 μm.

Y2H-based protein-interaction tests were conducted between FcWD40-97 and FcMYBs/FcbHLHs. Yeast cells of co-transformants with four combinations: BD–FcWD40-97 plus AD–FcMYB114, BD–FcWD40-97 plus AD–FcMYB123, BD–FcWD40-97 plus AD–FcbHLH1, BD–FcWD40-97 plus AD–FcbHLH42, BD–FcWD40-97 plus AD–FcMYC2 grew on the DDO medium. Moreover, they also grew on QDO/X-α-gal/AbA and the colonies turned blue (Figure 8A).

Three anthocyanin biosynthesis-related proteins were selected for further validation by BiFC. Yellow fluorescent protein (YFP) fluorescence was observed in the nuclei of tobacco leaf cells co-transformed with YCE–FcWD40-97 plus YNE–FcMYB114, YCE–FcWD40-97 plus YNE–FcMYB123, and YCE–FcWD40-97 plus YNE–FcbHLH42 (Figure 8B). No YFP fluorescence was observed in the control combinations, i.e., empty YNE vector plus YCE– FcWD40-97, and empty YCE plus YNE–FcMYB114, YNE–FcMYB123, or YNE–FcbHLH42. The results demonstrated that FcWD40-97 interacts with FcMYB114, FcMYB123, and FcbHLH42 *in vivo*, and is potentially able to form the MBW transcription complex in fig. This confirmed the identity of *FcWD40-97* as *FcTTG1*.

## Discussion

### *FcWD40* Family

There are around 200 putative WD-containing proteins in diploid plants which serve important roles in a wide spectrum of physiological functions, such as growth, the cell cycle, signal transduction, chromatin remodeling, transcriptional regulation, and others. WD40 domain-containing proteins have also been identified in prokaryotes, as well as lower eukaryotes. It has been postulated that the WD40 repeats arose from intragenic duplication and recombination events (Andrade et al., 2001); however, recently, the common ancestry of WD40 genes has been questioned (Jain and Pandey, 2018).

Tandem and segmental duplications are primary driving forces in generating members of a gene family during evolution (Cannon et al., 2004). In our study, 3 and 8 pairs of FcWD40s among the 204 WD40-containing proteins in fig were predicted as resulting from tandem duplications and segmental duplication events, respectively. This ratio is significantly lower than that of two other gene families that we previously studied: among 118 identified *FcbHLHs*, 11 and 4 pairs were predicted to originate from tandem replication and fragment replication, respectively (Song et al., 2021), and among 31 *FcPLCP* genes, 6 duplicated gene pairs were predicted (Zhai et al., 2021).

A similar frequency of tandem and segmental duplications has been predicted for *WD40* genes in other plants. Among the 230 *WD40* genes of *Arabidopsis*, 13 were identified as involved in tandem duplication events (Li et al., 2014). In the 178 *WD40* genes of potato, 3 pairs of genes were identified as tandemly duplicated, and 14 pairs of genes were related to segmental duplication (Liu et al., 2020). Among of the 187 *WD40* genes in rose, 11 members were identified as stemming from tandem duplication (Sun et al., 2020). In the hexaploid wheat, 743 *WD40* genes were identified, and 39 and 46 pairs of *WD40*s were distinguished as tandem and segmental duplication genes, respectively (Hu et al., 2018). In 184 and 206 *WD40* genes of *F. hispida* and *F. microcarpa*, 9 and 14 pairs of tandem duplications were identified, and 15 and 13 genes were related to segmental duplication, respectively (Zhang et al., 2020). In our study, all *FcWD40* tandem duplications originated from subfamily IV, and segmental duplication gene pairs were mainly from subfamily III. Our findings suggest tandem and segmental duplication as important driving forces for expansion in specific FcWD40 subfamilies, whereas they are less crucial in the evolution of the FcWD40 family as a whole.

### FcTTG1 Homologs and Expression Characteristics

Genes with similar amino acid sequences usually have similar functions, and vice versa (Liu et al., 2022). Eight TTG1 proteins from different plants were found to be 78.2%–99.12% homologous, indicating that the functions of TTG1s are conserved in different species. In the present study, FcWD40-97 showed the highest similarity to TTG1s of other plants, with a deduced amino acid sequence that had 79.88% similarity to AtTTG1 (Li et al., 2014), 88.01 and 87.94% to apple TTG1 and peach WD40, respectively (An et al., 2012; Cui et al., 2021), 85.67, 85.17, and 85.59% to CsWD40, FaTTG1, and *Punica granatum* (Pg) TTG1, respectively (Ben-Simhon et al., 2011; Schaart et al., 2013; Liu et al., 2018), and 82.90 and 80.52% to G*ossypium hirsutum* (Gh) TTG3 and *Medicago truncatula* (Mt) WD40-1 (Humphries et al., 2005; Pang et al., 2009), respectively. Moreover, FcWD40-97 shared the four highly conserved WDR motifs with other TTG1s, reported to be related to anthocyanin biosynthesis. Therefore we suggest that *FcWD40*-97 is *FcTTG1.*

*FcTTG1* encoded 339 amino acids, which is more than grape TTG1 (336), but less than TTG1 of *Arabidopsis* (341), strawberry (344) and *Camellia sinensis* (342). In addition, FcTTG1 has 5 WDR motifs, similar to strawberry (Schaart et al., 2013), tea tree (Liu et al., 2018), grape (Jiu et al., 2021) and pear (Cui et al., 2021), whereas *Arabidopsis* (Baudry et al., 2004), Chinese bayberry (Liu et al., 2013) and pomegranate (Ben-Simhon et al., 2011) each have 4 WDR motifs.

Many studies have reported that *TTG1* genes are mainly expressed in anthocyanin-accumulating tissues. Apple *TTG1* showed the highest transcript level in the peel (An et al., 2012). The expression level of *PgWD40* was higher in red-colored leaves and stems than in their green-colored counterparts (Ben-Simhon et al., 2011). Chinese bayberry *WD40-1* showed the highest transcription level in fruit (Liu et al., 2013). *Freesia* × *hybrida TTG1* was mainly expressed in petals (Shan et al., 2019). In our study, *FcTTG1* was preferentially expressed in fruit flesh and peel, and highest expression in these two tissues was found at developmental stage 4. The expression pattern in fig flesh was in line with the high expression of *FcMYB114* and *FcbHLH42*, which are the major transcription regulators in fig pigmentation (Song et al., 2021); in fig peel, *FcTTG1* was highly expressed before *FcMYB114* and *FcbHLH42.*

### Conservation and Pleiotropy of FcTTG1 in the Isoflavone-Biosynthesis Pathway

The TTG1-dependent MBW transcriptional complex is necessary for the regulation of anthocyanin/proanthocyanidin biosynthesis among plant species (Lloyd et al., 2017). In *Arabidopsis*, the *ttg1* mutant lacks seed-coat pigmentation, resulting in a transparent testa (tt) phenotype; absence of anthocyanin biosynthesis was also observed in the epidermis and subepidermal layers of *ttg1* mutant leaves and stems (Walker et al., 1999). The rice homozygous *ttg1* mutant exhibits significantly decreased anthocyanin accumulation in various organs (Yang et al., 2021), validating the crucial role of TTG1 in the MBW complex.

A small group of MYBs and bHLHs have been identified as components of the MBW complex. In *Arabidopsis*, PAP1, PAP2, MYB113, MYB114, and TT2 are MYBs that regulate anthocyanin and proanthocyanidin biosynthesis (Nesi et al., 2001; Gonzalez et al., 2008). In apple, MYB1, MYBA, MYB3, MYB110a, and MYB10 regulate anthocyanin biosynthesis in the peel, flesh and leaves (Ban et al., 2007; Espley et al., 2007; Chagné et al., 2013; Vimolmangkang et al., 2013), while MYB23 regulates proanthocyanidin biosynthesis (An et al., 2018). In grape, MYBA1, MYBA2, MYBA5, MYBA6, and MYBA7 were identified as regulators of anthocyanin biosynthesis (Matus et al., 2017), while MYBPAR regulates proanthocyanidin biosynthesis (Koyama et al., 2014). Similar redundance has been found with bHLHs in anthocyanin/proanthocyanidin biosynthesis. *Arabidopsis* bHLHs GL3, EGL3, and TT8 were reported as components of MBW in flavonoid-biosynthesis regulation (Nesi et al., 2001; Gonzalez et al., 2008; Xu et al., 2015).

In contrast to the small group of MYBs and bHLHs involved in isoflavone biosynthesis, less complexity has been found with TTG1. TTG1 demonstrates high conservation and pleiotropy in different MBW complexes. In our study, FcTTG1 could interact with FcMYB114, FcMYB123, and FcbHLH42, indicating the generality of FcTTG1 in fig anthocyanin and proanthocyanidin biosynthesis. Similarly, strawberry *TTG1* was reported to control proanthocyanidin biosynthesis together with *FaMYB9*/*FaMYB11* and *FabHLH3* (Schaart et al., 2013). In mango, TTG1 was found to interact with MYB0, TT8 and bHLH1 (Tan et al., 2021).

The constitutive nature of FcTTG1 in the MBW complex is further supported by its expression pattern under different light treatments. *FcTTG1* expression was not significantly changed when fig peel anthocyanin accumulation was almost completely inhibited by light deprivation, in line with the rice wild-type *TTG1* which is not significantly affected by light vs. dark (Yang et al., 2021). Nevertheless, light deprivation led to 1.59-fold downregulation of *FcMYB114* and 0.82-fold downregulation of *FcbHLH42* in the peel (Wang et al., 2019; Song et al., 2021). Similarly, *Arabidopsis TTG1* is rather stably expressed in both seedlings and rosette leaves when treated with white light, whereas *MYBs* (*PAP1* and *PAP2*) and *bHLHs* (*TT8*, *EGL3* and *GL3*) are upregulated (Cominelli et al., 2008).

Similar to other TTG1s, FcTTG1 serves as an essential and constitutive component of MBW complexes, that is, variable R2R3–MYBs and bHLHs share the same TTG1 constituents. The tissue-specific pattern of anthocyanin and proanthocyanidin biosynthesis and the response to environmental stimuli rely more on R2R3–MYB factors, whereas TTG1 is an indispensable and constant regulator in the MBW complexes.

## Conclusion

We identified 204 *FcWD40* genes in the fig genome, and described their chromosome distribution, evolutionary characteristics, gene structure, and cellular localization. The expression patterns of *FcWD40*s during fig fruit development and in response to light deprivation were grouped. Agglomerative hierarchical clustering recruited FcWD40-97 as the ideal candidate FcTTG1, as further confirmed by a set of molecular biology validations. FcWD40 was revealed as a constitutive component of the MBW complex regulating anthocyanin biosynthesis of fig fruit. This result uncovers the specific FcWD40 in fig anthocyanin biosynthesis and extends our knowledge on fig fruit color development. Further functional studies should be performed to confirm the role of FcTTG1 in fig fruit development.

## Data Availability Statement

The datasets presented in this study can be found in online repositories. The names of the repository/repositories and accession number(s) can be found below: PRJNA723733 and PRJNA494945.

## Author Contributions

ZF, YZ, and YW performed the experiments. LZ analyzed the data. ZF prepared the first draft of the manuscript. MF and HM revised the manuscript. HM supervised the manuscript. All authors contributed to the article and approved the submitted version.

## Conflict of Interest

The authors declare that the research was conducted in the absence of any commercial or financial relationships that could be construed as a potential conflict of interest.

## Publisher’s Note

All claims expressed in this article are solely those of the authors and do not necessarily represent those of their affiliated organizations, or those of the publisher, the editors and the reviewers. Any product that may be evaluated in this article, or claim that may be made by its manufacturer, is not guaranteed or endorsed by the publisher.

## Footnotes

1
https://www.ncbi.nlm.nih.gov/Traces/wgs/VYVB01?display=history%EF%BC%89

2
http://arabidopsis.org

3
http://pfam.xfam.org/family/PF00400

4
http://hmmer.org/

5
https://www.ncbi.nlm.nih.gov/cdd/

6
http://web.expasy.org/compute_pi/

7
https://psort.hgc.jp/form2.html

8
https://sourceforge.net/projects/kakscalculator2/

9
https://www.ncbi.nlm.nih.gov/bioproject/PRJNA723733

10
https://www.ncbi.nlm.nih.gov/bioproject/?term=PRJNA494945

11
http://deweylab.biostat.wisc.edu/rsem/

12
http://www.bioconductor.org/packages/2.12/bioc/html/edgeR.html

13
https://cn.string-db.org/cgi/network?taskId=bvuGEO8PZi4D&sessionId=b9QKtOaUD9B4

## Supplementary Material

Supplementary Figure 1 Collinearity analysis of *FcWD40* genes; circle plot created with MCScanX tool. Identified collinear genes are linked by colored lines.

Supplementary Figure 2 Phylogenetic analysis of 66 FcWD40s with predicted nuclear localization and at least 4 WD-repeat (WDR) motifs.

Supplementary Figure 3 Phylogenetic analysis of FcWD40s and TTG1s of *Arabidopsis thaliana*, *Malus domestica*, *Pyrus pyrifolia*, *Fragaria* × *ananassa*, *Camellia sinensis*, *Punica granatum*, G*ossypium hirsutum*, and *Medicago truncatula*. *FcWD40-97* is indicated with red dot. Blue color background indicates TTG1 subgroups, and red star indicates potential TTG1 homolog in fig.

Supplementary Figure 4 Interaction of control combinations *in vivo*.
