# Supplementary figures and images for: Genome-Wide Analysis of Anthocyanin Biosynthesis Regulatory WD40 Gene FcTTG1 and Related Family in Ficus carica L
Source: Front Plant Sci. 2022 Jul 14;13:948084. doi: 10.3389/fpls.2022.948084 (PMC9334019; doi:10.3389/fpls.2022.948084)

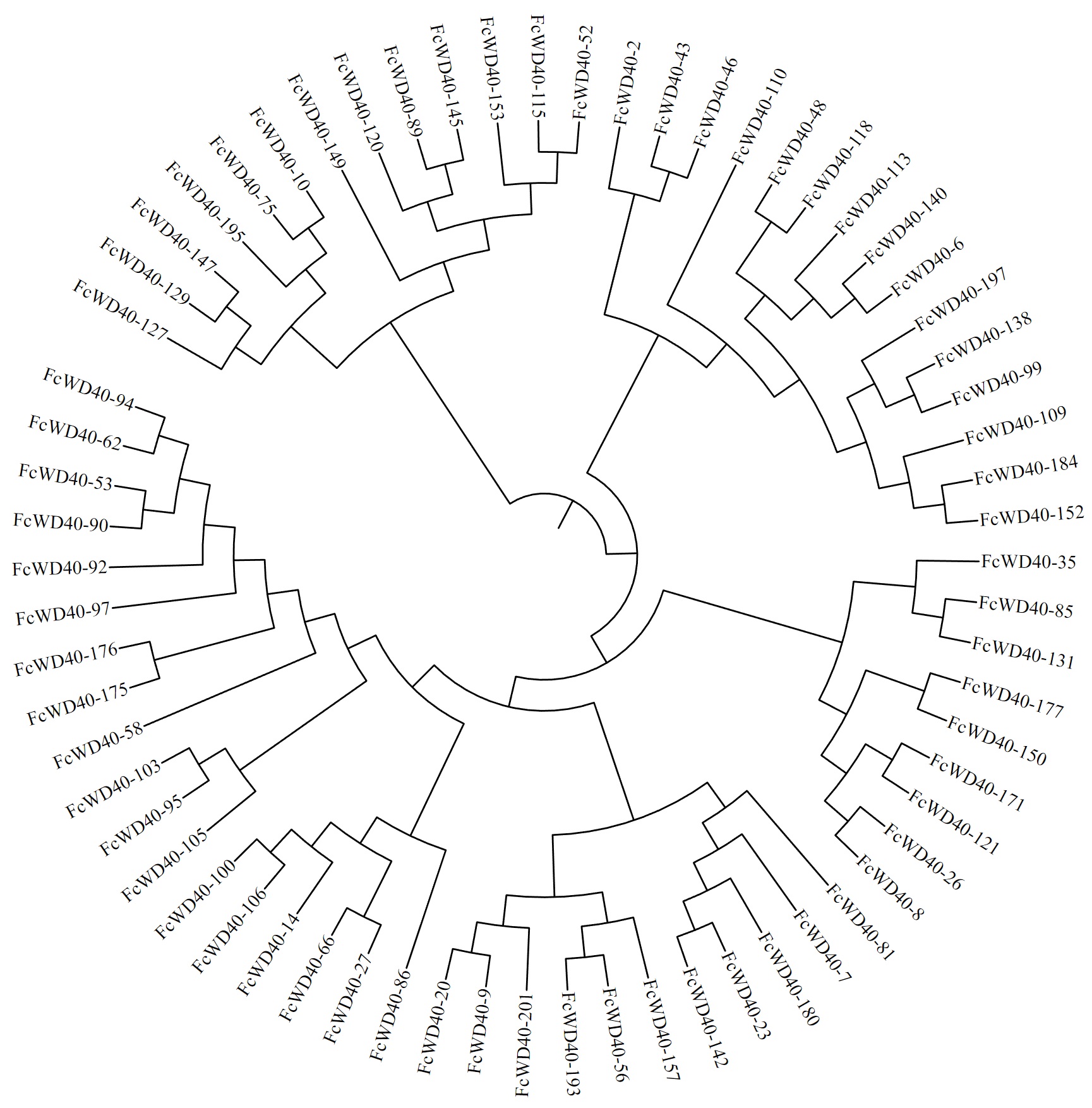

Supplement: Supplementary Figure 2 — Phylogenetic analysis of 66 FcWD40s with predicted nuclear localization and at least 4 WD-repeat (WDR) motifs. [file Image_2.JPEG]

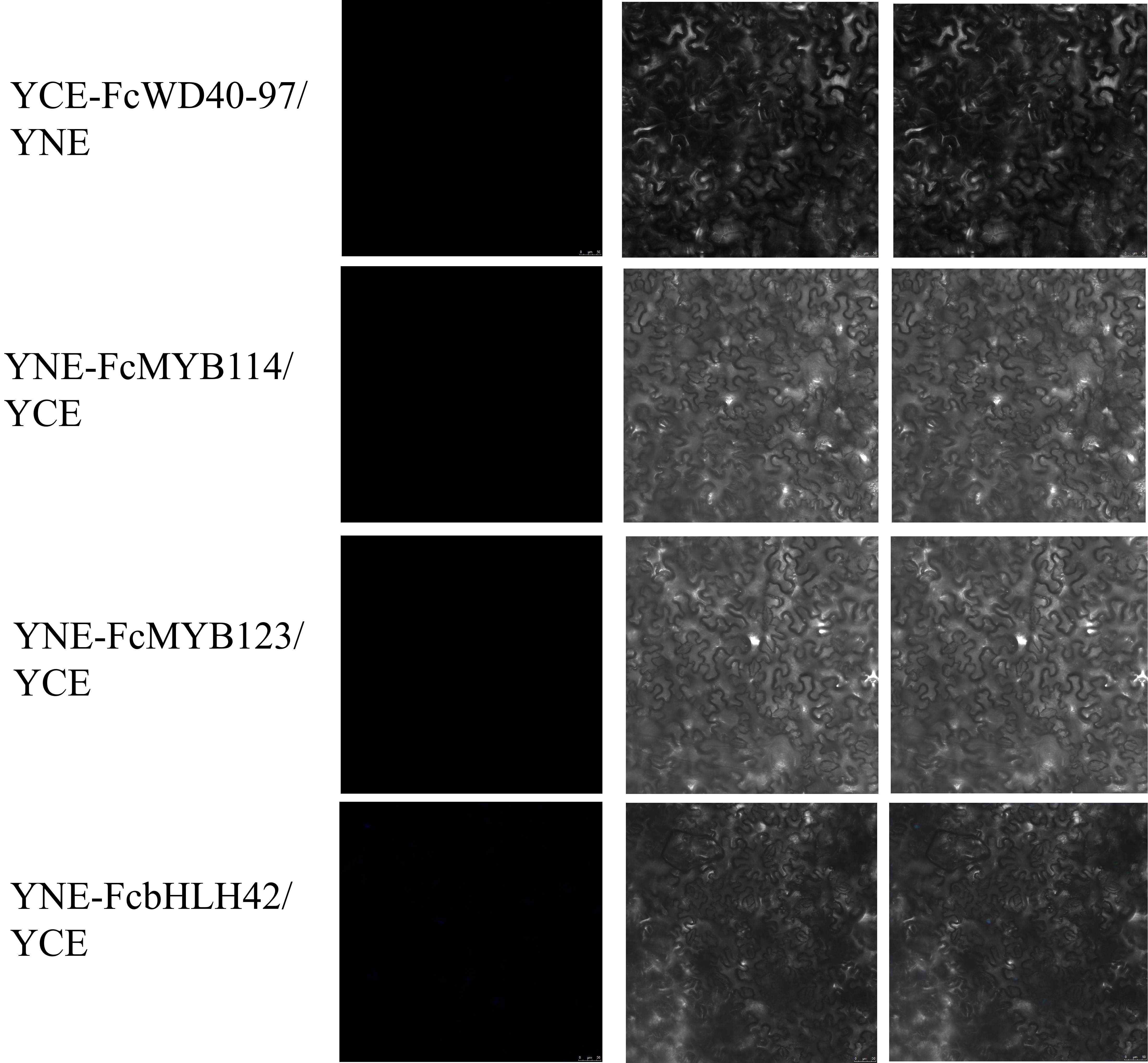

Supplement: Supplementary Figure 4 — Interaction of control combinations in vivo. [file Image_4.JPEG]
